# Synthesis, structural, molecular docking, and in vitro biological activities of Cu-doped ZnO nanomaterials

**DOI:** 10.1038/s41598-024-59088-2

**Published:** 2024-04-19

**Authors:** Ahmed F. El-Sayed, Wael M. Aboulthana, Marwa A. Sherief, Gehan T. El-Bassyouni, Sahar M. Mousa

**Affiliations:** 1 Microbial Genetics Department, Biotechnology Research Institute, National Research Centre, 33 El Bohouth St. (Former El Tahrir St.), P.O. 12622, Dokki, Cairo, Egypt; 2 Egypt Center for Research and Regenerative Medicine (ECRRM), Cairo, Egypt; 3 Biochemistry Department, Biotechnology Research Institute, National Research Centre, 33 El Bohouth St. (Former El Tahrir St.), P.O. 12622, Dokki, Cairo, Egypt; 4 Inorganic Chemistry Department, Advanced Materials Technology and Mineral Resources Research Institute, National Research Centre, 33 El Bohouth St. (Former El Tahrir St.), P.O. 12622, Dokki, Cairo, Egypt; 5 Refractories, Ceramics and Building Materials Department, Advanced Materials Technology and Mineral Resources Research Institute, National Research Centre, 33 El Bohouth St. (Former El Tahrir St.), P.O. 12622, Dokki, Cairo, Egypt

**Keywords:** Cu-doped ZnO, Antioxidant, Antimicrobial, Anti-diabetic, α-amylase, Electrophoresis, Docking, Enzymes, Biochemistry, Origin of life

## Abstract

Copper-doped ZnO nanoparticles with the formula Zn_1−x(Cu)_O, where x = 0.0, 0.03, 0.05, and 0.07 were produced using the co-precipitation process. Physical, chemical, and structural properties were properly examined. Powdered X-ray diffraction (P-XRD) patterns revealed the formation of hexagonal wurtzite crystal structure in all samples, through atomic substitutional incorporation in the Cu-doped ZnO lattice. The presence of Cu ions and their dissolution in the host ZnO crystal structure was supported by FT-IR spectra. HR-TEM images were used to assess the average size, morphology, and shape regularity of the synthesized samples. The form and homogeneity of the ZnO changed when Cu ions were substituted, as evidenced by FE-SEM/EDX analysis. The presence of copper signals in the Cu-doped samples indicates that the doping was successful. The decrease in zeta potential with an increased copper doping percentage designates that the nanoparticles (NPs) are more stable, which could be attributed to an increase in the ionic strength of the aqueous solution. The synthesized NPs were evaluated for their substantial in vitro antioxidant properties. In addition, the antimicrobial efficacy of the materials was tested against pathogenic microorganisms. Regarding the anti-diabetic activity, the 7Cu ZnO sample showed the highest inhibitory effect on the α-amylase enzyme. No variations were observed in the activities of the acetylcholinesterase enzyme (AChE) and proteinase enzymes with ZnO and samples doped with different concentrations of Cu. Therefore, further studies are recommended to reveal the in-vitro anti-diabetic activity of the studied doped samples. Finally, molecular docking provided valuable insights into the potential binding interactions of Cu-doped ZnO with α-amylase, FabH of *E. coli*, and Penicillin-binding proteins of *S. aureus*. These outcomes suggest that the prepared materials may have an inhibitory effect on enzymes and hold promise in the battle against microbial infections and diabetes.

## Introduction

Nanometal oxides find wide-ranging applications in fields such as gas sensors, batteries, fuel cells, microelectronic circuits, and high-temperature superconductors. They can be used as catalysts, sorbents, and antibacterial agents, among other things, because of their large surface area and small size. Additionally, these characteristics enhance electrical characteristics, conductivity, and catalytic activity^1^.

Antibacterial qualities of photocatalytic materials are effective against multidrug-resistant bacteria, including *E. coli, S. aureus, P. aeroginosa, S. mutants*, and *K. pneumonia*^2^. Bacterial cell membranes contain nanoscale pores, and the synthesized nanoparticles (NPs) can penetrate these pores to exert an antagonistic effect.

Zinc oxide (ZnO) is a photocatalytic material that is used in various fields of study, including catalysis, sensors, antibacterial and antifungal treatments^3,4^ and biomedical applications, due to its high stability, exceptional crystallinity, and saturation velocity^5^.

Due to the large energy band gap of ZnO (3.3 eV), many studies have been conducted to minimize the band gap of ZnO to improve the photocatalytic activity. These studies embrace precious metal deposition^6^, doping metal ions^7^, composite semiconductors^8^, and non-metallic doping^9^. Doping engineering in nanotechnology alters critical metal oxide characteristics. Furthermore, the efficacy of the photocatalyst as an antimicrobial agent can be enhanced through the doping process. Various transition metal ions were added to host ZnO crystals to improve the physical characteristics and utility of ZnO nanoparticles (ZnO-NPs)^10,11^.

Doping of ZnO-NPs with appropriate transition metal ions within the percolation limit permits them to perform better in biomedical and other current applications^12^. Cu^2+^ dopant has a stronger influence on the microstructure of ZnO-NPs than the other transition metal ions. Cu^2+^ ions are easily absorbed into host ZnO nanocrystals due to their nontoxicity, almost identical ionic radii, and charge valence. Earlier, researchers revealed that Cu could dope ZnO up to 3 wt%, but at higher concentrations, composites of ZnO-CuO were formed, with two different phases comprising flake-like shaped elongated particles^13^.

Several techniques to produce pure Zn and Cu-doped ZnO were applied, such as the co-precipitation method^14,15^, sol–gel process^16^, chemical vapor deposition^17^, thermal decomposition^18^, hydrothermal synthesis^19^, and solid-state reaction^20^.

The co-precipitation approach, which has various benefits, was employed in this work to create both doped and undoped ZnO-NPs. Simplicity and affordability are two main reasons for the co-precipitation method that is advantageous for producing doped ZnO-NPs:^21^. This study aimed to synthesize and characterize the structure of Cu-doped ZnO nanomaterials. Subsequently, a thorough assessment of the biological activities of the materials was conducted, covering aspects such as antioxidant capacity, antimicrobial effectiveness, anti-diabetic effects, anti-diabetic properties, anti-Alzheimer's effects, and anti-inflammatory activity.

## Materials and methods

### Synthesis of pure ZnO and doped samples

To produce ZnO-NPs, deionized water was used to prepare 500 mL of an aqueous 0.05 M zinc acetate solution [Zn(CH_3_COO)_2_. 2H_2_O, Qualikems Reagent, 99%] and 50 mL of an aqueous 0.1 M NaOH solution [Merck, 99%]. At 50 °C, a bright translucent white gel formed when the NaOH solution was gradually added to the zinc acetate solution while being magnetically agitated for an hour. The mixture was left to stand at room temperature for a whole day. The supernatant was removed after centrifuging the solution for ten minutes at 3500 rpm. The precipitate was finely ground in a mortar after being dried at 150 °C for five hours. Zinc oxide doped with Cu samples (Zn_1−x_Cu _x_ O) were prepared similarly to the pure zinc oxide. The solution of zinc acetate was doped with the calculated amounts of copper acetate [Cu(CH_3_COO)_2_, Aldrich, 98%] where the Zn/(Zn + Cu) atomic ratio (denoted as xCu) varied between 0.03, 0.05 and 0.07. The remaining steps were the same as described above for the preparation of pure ZnO powder. The prepared samples were coded based on the doping concentration of copper ions, as illustrated in Table 1.

**Table 1:** Utilized symbols of ZnO doped with copper ions and their meanings.

Sample	Meaning
ZnO	Pure Zinc Oxide
3Cu ZnO	ZnO doped with 3% Cu
5Cu ZnO	ZnO doped with 5% Cu
7Cu ZnO	ZnO doped with 7% Cu

### Characterization of prepared materials

To determine the crystalline phases of the powdered materials before and after Cu doping; Powdered X-ray diffraction pattern (P-XRD) of type BRUKER, D8 ADVANCED, Cu target, Germany, fitted with an X-ray tube [CuK_α_ radiations: λ = 1.5406 Å] via Ni filter was used. The diffractometer was set at 40 kV, 40 mA, with a scan speed of 2°/min. The data was collected in the range of 2θ = 20–80°. The achieved phases were identified using the Joint Committee on Powder Diffraction Standard (JCPDS). The Debye-Scherer equation was used to calculate the crystallite size (Cs)^22^.

$$Cs = \, \left( {K\lambda /\beta \cos \theta } \right)$$

where, K is Scherrer’s constant (k = 0.94), λ is the X-ray wavelength, β is the full width at half maximum (FWHM) of the diffraction peak, and θ refers to the diffraction angle.

FT-IR analysis was used at room temperature to investigate the structural and compositional properties of powdered materials. The investigation was conducted utilizing an FT-IR spectrometer (Type A FTIR-6100, JASCO, USA) with a vibrational wave number range of 500–2500 cm^-1^ and a spectral resolution of 4 cm^−1^. To prepare the samples for qualitative analysis, the powdered materials were mixed with potassium bromide (KBr) in a 1:100 ratios. This combination was subsequently compacted into discs with a load of 5 tons/cm^2^ in an evocable die.

The microstructure and surface morphology of the processed samples were examined using a field-emission scanning electron microscope (FE-SEM) at 15 kV (Quanta 250 FEG, FEI, Netherlands). Gold was used to coat the sample surfaces, ensuring a clear morphological study. The coating procedure was carried out using a sputtering coater (S150A Edwards, England) with a vacuum of 0.1 Torr, a current of 50 mA, and a voltage of 1.2 kV.

The nature and crystallinity of nanoparticles were investigated with a high-resolution transmission electron microscope (HR-TEM) model JEM-2100 from Joel in Japan. The particles were drop-cast into an aqueous dispersion on a carbon-coated copper grid, then air-dried at room temperature before being examined microscopically.

### Evaluation of the in vitro biological activities

#### Antioxidant activity

The Total Antioxidant activity (TAC) was determined in mg gallic acid/g by analyzing the green phosphate/Mo^5+^ complex at a wavelength (λ) of 695 nm, following the procedure described by Prieto et al.^23^. 0.1 mL aliquots of samples were combined with 1 mL of reagent solution containing 0.3 N sulfuric acid, 28 mM sodium phosphate, and 4 mM ammonium molybdate. Methanol (80%) was used in place of the sample for the blank. The tubes were sealed and incubated for 90 min in a boiling water bath. After allowing cooling to ambient temperature, the absorbance was measured at 695 nm against the blank. The antioxidant capacity was expressed as mg gallic acid equivalent per gram dry weight. All samples were tested in triplicate.

The iron reduction power was measured in μg/mL using the method provided by Oyaizu^24^, employing ascorbic acid as the standard. In summary, 1 mL of the tested sample (1 mg/mL) was mixed with 1 mL of 200 mM sodium phosphate buffer (pH 6.6) and 1 mL of 1% potassium ferricyanide. The blend was then incubated at 50 °C for 20 min before being treated with 1 mL of trichloroacetic acid (10%). After centrifugation at 2000 rpm for 10 min, the upper layer solution (2.5 mL) was mixed with 2.5 mL of double deionized water and 1 mL of fresh ferric chloride (0.1%). The absorbance was measured at 700 nm against a blank prepared without the extract. Ascorbic acid in varied concentrations was utilized as a standard. A high absorbance at 700 nm indicates a higher reducing power in the reaction mixture.

#### Scavenging activity

The 2,2-diphenyl-1-picrylhydrazyl (DPPH) free radical scavenging performance was evaluated using the technique published earlier by Forootanfar et al.^25^. This procedure involved preparing three quantities of Cu and Zn NPs (500, 1000, and 1500 µg/mL) were prepared. In each tube, 3 mL of a freshly made methanolic DPPH solution with a concentration of 0.01 M was added. Each solution was well shaken and then stored in the dark for a period of one hour. Following the incubation period, the absorbance of each solution combination was measured at a wavelength of 517 nm^26^. To assess the antioxidant activity of each dilution of Cu and ZnO-NPs, the ABTS [2,2'-azino-bis(3-ethylbenzothiazoline-6-sulfonic acid)] radical scavenging assay technique described by Re et al.^27^ was utilized. To perform this test, dilute the ABTS solution with methanol to attain an absorbance of 0.70 (± 0.02) at 734 nm. The solution was then equilibrated at 30 °C. For the assay, 1.0 mL of the diluted ABTS solution was mixed with 1 mL of each dilution containing the Cu and ZnO-NPs. The absorbance at 734 nm was measured exactly 30 min after mixing with a solvent blank at 30 °C. The percentage inhibition was estimated using the following formula:

$${\text{Scavenging}}\;{\text{activity }}\left( \% \right) \, = \, \left[ {1 - \, \left( {\frac{{\text{absorbance of sample}}}{{\text{absorbance of control}}}} \right) \, \times \, 100\% } \right].$$

#### Anti-diabetic activity

##### Enzymatic assay

This assay involved estimating the inhibition percentages (%) of α-amylase enzyme using the procedure demonstrated by Wickramaratne et al.^28^ with Acarbose as the standard drug. During the assay, 0.5 mL of the test solution was mixed with 0.5 mL of α-amylase solution (0.5 mg/mL) and buffer (Na_2_HPO_4_/NaH_2_PO_4_ (0.02 M), NaCl (0.006 M) at pH 6.9) to achieve concentrations ranging from 25 to 800 μg/mL. After waiting 10 min at room temperature, 200 μL of starch solution (1% in water (w/v) buffer [Na_2_HPO_4_/NaH_2_PO_4_ (0.02 M), NaCl (0.006 M) at pH 6.9)] was added. To stop the reaction, add 200 μL of DNSA (coloring) reagent (12 g of sodium potassium tartrate tetrahydrate in 8.0 mL of 2 M NaOH and 20 mL of 96 mM DNSA solution). The test tubes were then placed in a boiling water bath (100 °C) for 10 min, after which the mixture was cooled to room temperature and diluted with 5 mL of distilled water. A UV–Visible spectrophotometer set to 540 nm was used to measure the absorbance. The median inhibitory concentration (IC_50_) of each tested sample was derived by constructing a curve using a series of sample concentrations (1, 2, 3, 4, 5, and 6 mg/mL) vs the percent of α-amylase inhibition.

##### Electrophoretic α-amylase pattern

This assay utilized polyacrylamide gel electrophoresis (PAGE) according to the method proposed by Rammesmayer and Praznik^29^*.* Following the electrophoresis run, the native gel was dissected and washed with Tris–HCl buffer (pH 7.1) and incubated with working buffer containing 50 mL of Tris–HCL (pH 7.5) (6 g /1L), 110 mg CaCl_2_ and 0.5 g soluble starch. To visualize the electrophoretically separated α-amylase types, they were incubated in a staining solution containing Pot. Iodide (300 mg) and iodine (130 mg) dissolved in 100 mL distilled water. The PAGE plate was photographed, and the native isoenzyme pattern was evaluated using the Quantity One software programe (version 4.6.2) to govern the relative mobility (Rf), band quantity (Qty) and band percent (B%) of the electrophoretically separated bands. The proportions of similarity index (SI%) and physiological difference (Diff%) were calculated using Nei and Li's equation^30^.

#### Anti-Alzheimer's activity

In this investigation, we used Ellman's method^31^ to determine the percentage of inhibition of the acetylcholinesterase (AChE) enzyme, with donepezil as the standard drug. The tested sample was dissolved in a 0.1 M phosphate buffer at pH 8. For each run, 5 µL of acetylthiocholine (ATCh) at a concentration of 0.5 mM, 5 µL of 5,5’-dithiobis-2-nitrobenzoic acid (DTNB) at a concentration of 0.03 mM, and 5 µL of the tested sample solution at different concentrations were set to a flat bottom 96-well plate. The mixture was then incubated for 10 min at 30 °C. After incubation, 5 µL of AChE at a concentration of 0.3 U/mL was added to initiate the reaction, and the absorbance was measured at 412 nm. A control run was likewise performed, with all components except the test extract. All experiments were done in triplicate.

#### Anti-inflammatory activity

This assay measured protein denaturation (%)^32^ and proteinase inhibition^33^ using diclofenac sodium as the standard non-steroidal anti-inflammatory drug, as produced by Meera et al.^34^. The protein denaturation (%) was determined by mixing 0.5 mL of the test control solution, which was made by combining 0.45 mL of bovine serum albumin (BSA) (5% w/v aqueous solution) with 0.05 mL of distilled water. The product control, 0.5 mL was created by combining 0.05 mL of the test solution with 0.45 mL of distilled water. The various samples (test solution) and diclofenac sodium (standard) were utilized. The pH of all solutions was adjusted to 6.3 with HCl (1N). All of the samples were incubated at 37 °C for 20 min before being increased to 57 °C for 3 min. Following chilling, 2.5 mL of phosphate buffer was added to the formed solutions. A UV–visible spectrophotometer was used to determine the absorbance at 416 nm. The degree of protein denaturation inhibition can be calculated. Proteinase inhibitory activity was assessed by mixing the test sample (1 mL) with a reaction mixture comprising 0.06 mg trypsin dissolved in 1 mL of 20 mM Tris HCl buffer (pH 7.4). The mixture was then incubated for 5 min at 37 °C, before adding 1 mL of casein (0.8% w/v). After an additional 20 min of incubation, 2 mL of perchloric acid (70%) was added to terminate the reaction. The turbid suspension was centrifuged, and the absorbance of the supernatant at 210 nm was measured compared to a blank of buffer. The proteinase inhibitory activity was then calculated as a percentage.

#### Statistical analysis

A One-way analysis of variance (ANOVA) with the Statistical Package for the Social Sciences (SPSS for Windows, version 11.0) was performed to investigate statistically significant positive and negative associations between biological activity measurements. A significance level of p-value was used to determine significant correlations, with a "P" value less than 0.05 considered significant.

#### Antimicrobial activity

The strains used in this investigation were obtained from the Egyptian Microbial Genetics Lab at the National Research Centre. To assess the antibacterial activity of the prepared samples, agar diffusion was used to test a variety of gram-positive bacteria (*Staphylococcus aureus and Streptococcus mutants*) and gram-negative bacteria (*E. coli, P. aeruginosa, and K. pneumonia*). A bacterial culture was cultured on the solidified agar medium, and 1000 μg/mL of the prepared Cu and ZnO-NPs were added to the plates. After that, the plates were incubated at 37 °C for 24 h. The inhibition zone of bacterial growth was evaluated following the incubation period^35^.

### Molecular docking simulation

To acquire more insights and validate the observed biological activities, we investigated the activity of Cu-doped ZnO nanoparticles using molecular docking simulations. The RCSB Protein Data Bank was used to download anti-diabetic receptors (PDB: 2QV4) and anti-microbial protein targets (PDB: 5BNM for E. coli and 3HUM for S. aureus).

All crystal structures of the targets were prepared by removing water molecules, ions, and existing ligands using PyMOL software. Hydrogen atoms were then added to the receptor molecule using MG Tools of Autodock Vina software and saved into a dockable pdbqt format. According to Lattice parameter values of pure ZnO samples in Table 2 we used VESTA (Visualization for Electronic STructural Analysis), 3D visualization program for structural models, volumetric data, and crystal morphologies, as follow: (1) draw structure in CIF format (Crystallographic Information File), then, The cif format was converted to 3D Mol2 format and further converted using Open Babel to 3D PDB format. (3) The structure in PDB format was used as input for docking simulation. (4) Internal degrees of freedom were set to zero. (5) The structure was changed to the pdbqt format using Autodock tools. (6) The chemical structures were subjected to energy minimization^36^. Before docking, ligand-centered maps were generated by the AutoGrid program with grid dimensions of 90A˚ x 90A˚ x 90A˚. All docking experiments were accomplished using Autodock Vina 1.0 and the active site coordinates. Finally, analysis of the 2D interactions of the targets with the Cu-doped ZnO structure was performed using the Discovery Studio 4.5 program.

**Table 2:** Lattice parameter values of pure ZnO and doped samples with different concentrations of Cu.

Sample	Lattice parameters (Å)		Crystallite size (nm)
	a	c	
ZnO	3.2506	5.2069	35
3Cu ZnO	3.2496	5.2055	38
5Cu ZnO	3.2497	5.2056	42
7Cu ZnO	3.2507	5.2071	40

## Results and discussion

### Characterization of prepared samples

#### P-XRD analysis

**Figure 1:**
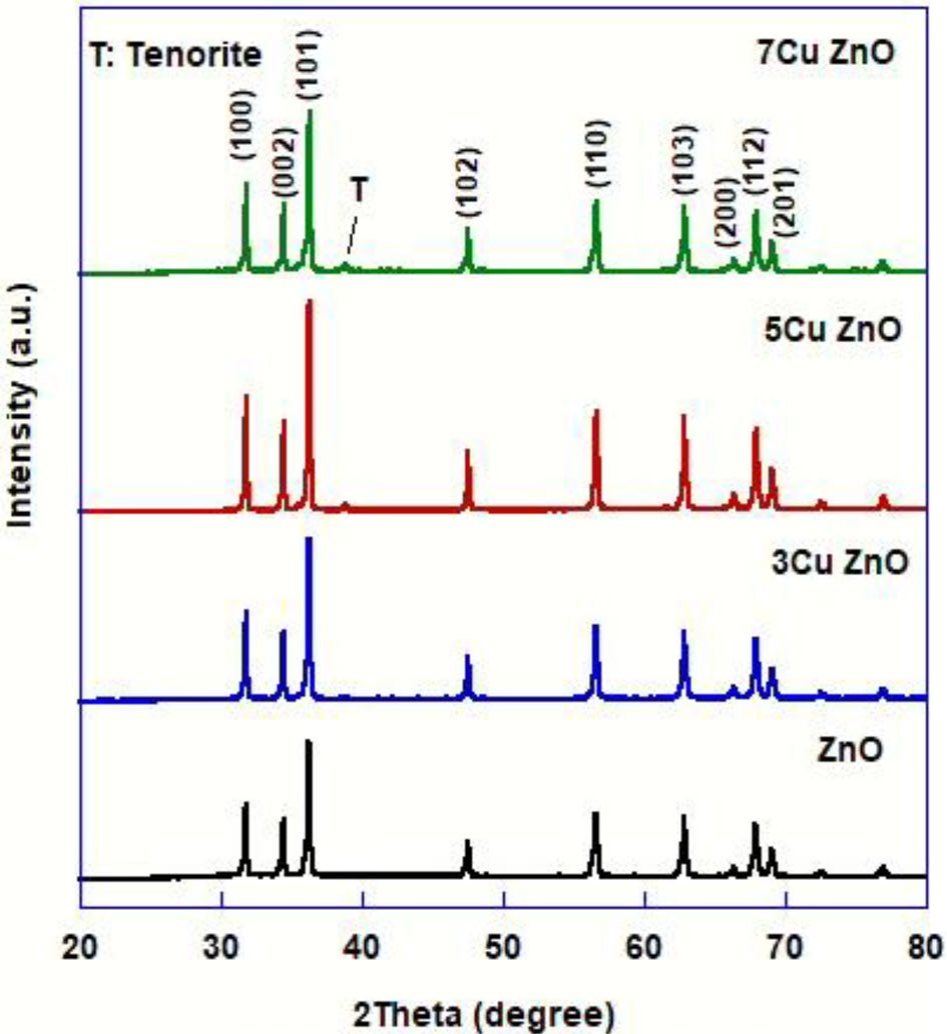
P-XRD patterns of pure ZnO and doped samples with different concentrations of Cu.

Figure 1 shows the P-XRD patterns of pure ZnO and samples doped with different Cu concentrations (3%, 5%, and 7%). The hexagonal structure of ZnO accounts for the preferred orientation around planes (100), (002), (101), (102), (110), (103), (200), and (112). Cu-doped ZnO has the same hexagonal wurtzite structure as pure ZnO. A small diffraction peak at 2θ = 39.5° is due to CuO (tenorite; JCPDS No. 41-0254). The change in lattice parameter values due to the doping process is presented in Table 2, which demonstrates negligible impact of CuO concentration up to 7 wt.% on the lattice parameter and crystallite size where the insertion of Cu^2+^ ions into the ZnO crystal has substantial influence on the lattice parameters (a and c) and crystallite size of ZnO because both Cu^2+^ ions and Zn^2+^ ions have similar ionic radii of 0.73 and 0.74 nm, respectively^37,38^.

#### FT-IR analysis

**Figure 2:**
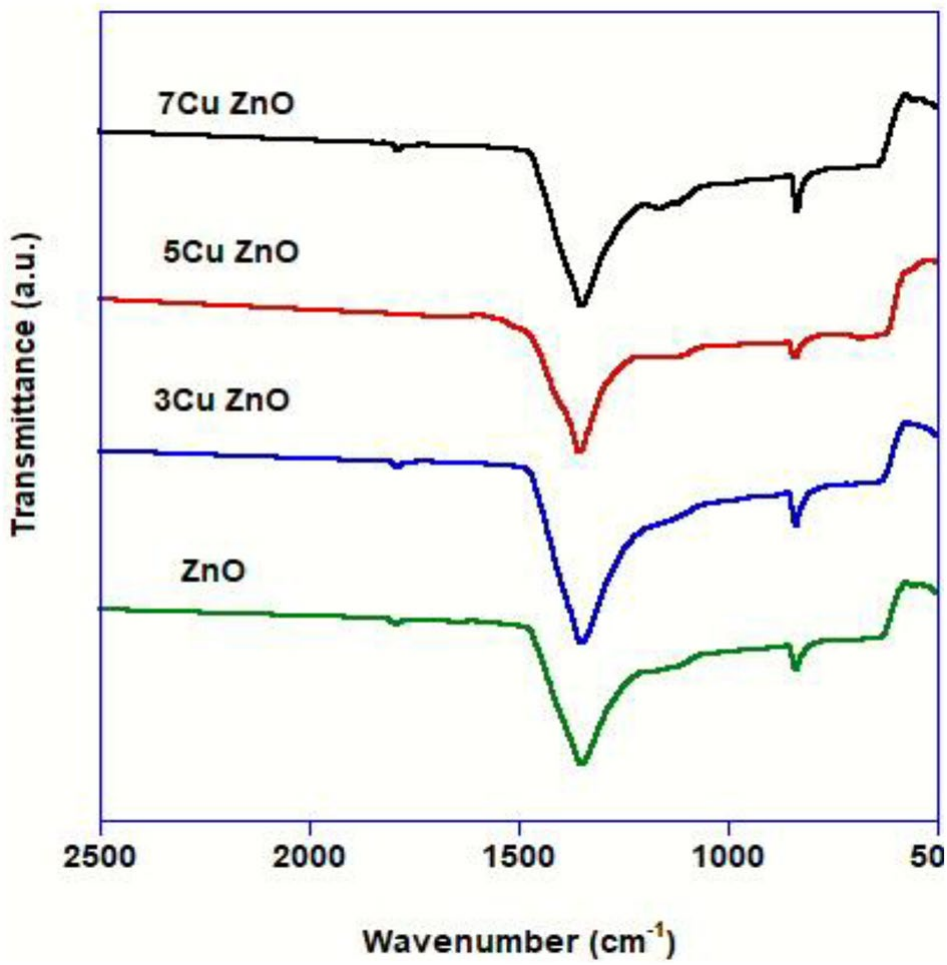
FT-IR spectra (with the horizontal scale from 2500 to 500) of pure ZnO and samples doped with different concentrations of Cu.

FT-IR spectra were utilized to investigate how doping ZnO-NPs with Cu^2+^ altered their vibrational properties. To investigate, the functional groups in the range of 2500–500 cm^-1^ region; FT-IR spectroscopy was performed (Fig. 2). The bands detected between 650 and 420 cm^-1^ were discovered to be the stretching mode of the metal–oxygen (M–O), which is linked to the vibrations of Cu–O and Zn–O, respectively. Two vibrational modes were seen in the infrared spectra at approximately 1350 and 840 cm^-1^, corresponding to the non-coordinate vibrational modes of the carbonate ion^39–41^. According to Ashwinia et al*.*^42^, the absence of -OH group suggested that no water had been adsorbed on the nano-crystalline powder's surface. Cu^2+^ ions were confirmed to be present in the ZnO's lattice sites by the FT-IR analysis result in a slight shift in the location of the absorption band, which might be attributed to variations in the bond length caused by the substitution of Cu^2^ for Zn^2+^.

#### FE-SEM/EDX

**Figure 3:**
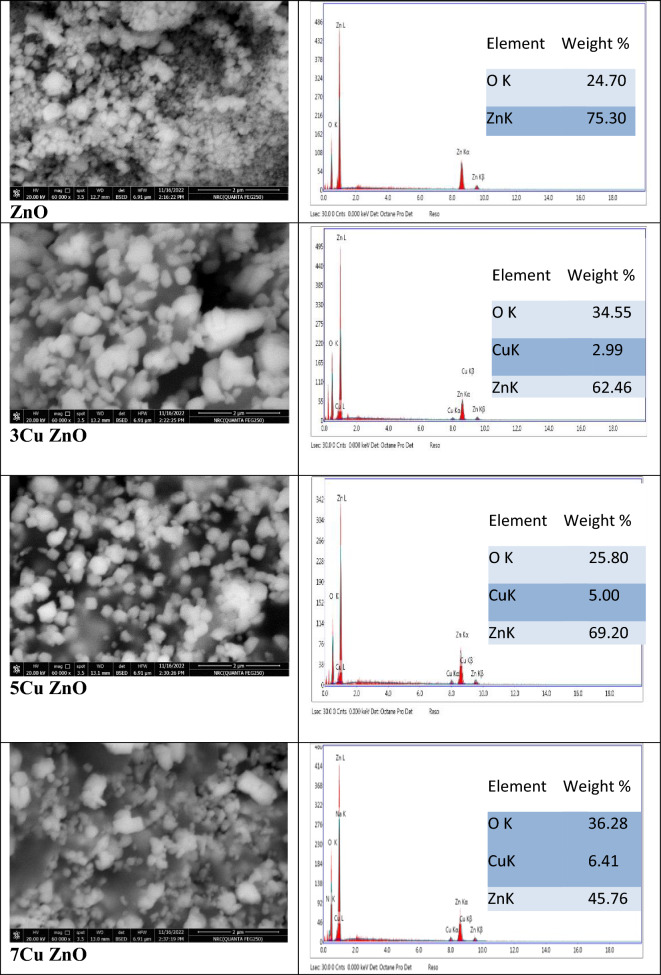
SEM with EDX of pure ZnO and doped samples with different concentrations of Cu.

FE-SEM was used to investigate the properties of both pure and Cu-doped ZnO. The images of the NPs are shown in Fig. 3. The grown-up NPs had a similar spherical size distribution to pure ZnO-NPs, which had a unique shape^43,44^. ZnO-NP formation was verified by elemental composition analysis using EDX, with a zinc/oxygen ratio ranging from 24.7 to 75.3 wt%. For doped samples, the shape and morphology of the NPs changed as the concentration of Cu increased due to the substitution of Cu^2+^ into the Zn sites^45^ where the particles were a collection of various shapes^46^. The images of doped samples also demonstrated that the agglomeration of nanoparticles increased with increasing Cu concentration^47,48^. The successful doping process was confirmed using EDX where copper signals were seen in the EDX patterns of doped samples and its intensity increased by increasing Cu concentration (Fig. 3).

#### HR-TEM

**Figure 4:**
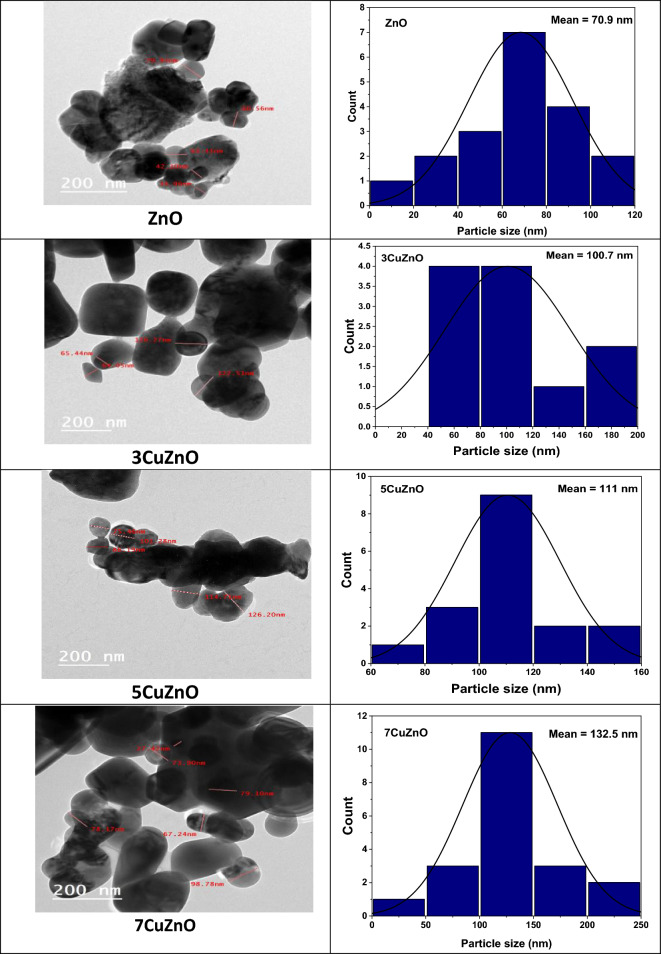
HR-TEM images of pure ZnO and Cu-doped samples and their corresponding histograms.

The incidence of NPs with an uneven size distribution and pseudo-spherical shape is exposed by the HR-TEM image of ZnO presented in Fig. 4. Because of their large surface area to volume ratio and high surface energy, ZnO nanoparticles have high ability to combine and aggregate, which is most likely the cause of the non-uniformity in particle size^49^. HR-TEM micrographs of Cu doped ZnO samples were composed of irregular microstructure and the agglomeration of nanoparticles increased by increasing Cu concentration^50^. The histograms (Fig. 4) showed the increasing of particle size as the concentration of Cu increased. The size distribution of nanoparticles was estimated by an image analyzer (Image J, 1.42 q developed by NIH Bethesda, Maryland, USA).

### In vitro* biological activities*

#### Antioxidant activity

The antioxidant activity of ZnO-NPs and samples doped with different concentrations of Cu (3, 5, and 7%) was measured by assaying TAC and IRP at three concentrations (0.5, 1, and 1.5 mg/mL). As depicted in Table 3, it was found that both TAC and IRP of monometallic ZnO increased with increasing concentration. Therefore, the ZnO at a concentration of 1.5 mg/mL possessed higher TAC (71.84 ± 0.06 mg gallic acid/g) and IRP (32.15 ± 0.05 µg/mL) compared to the other ZnO concentrations (0.5 and 1 mg/mL). This could be linked to the structural configuration of the oxygen atom, which controls the thermal stability of the nanoparticle based on its oxides' available free energy^51^. Moreover, the reducing power activity of ZnO-NPs was related to the presence of a metallic structure, which has the potential to contribute to the reduction reaction^52^.

**Table 3:** The antioxidant activity of ZnO-NPs and samples doped with different concentrations of Cu (3, 5, and 7%).

Sample	Concentration (µg/mL)	TAC (mg gallic acid/g)	IRP (µg/mL)
ZnO	500	25.21 ± 0.02	11.28 ± 0.02
	1000	46.63 ± 0.04	20.87 ± 0.03
	1500	71.84 ± 0.06	32.15 ± 0.05
3Cu ZnO	500	31.51 ± 0.03	14.10 ± 0.02
	1000	58.29 ± 0.05	26.09 ± 0.04
	1500	89.80 ± 0.07	40.19 ± 0.06
5Cu ZnO	500	38.76 ± 0.03	17.34 ± 0.03
	1000	71.70 ± 0.06	32.08 ± 0.05
	1500	110.45 ± 0.09	49.43 ± 0.08
7Cu ZnO	500	48.44 ± 0.04	21.68 ± 0.03
	1000	89.62 ± 0.07	40.11 ± 0.06
	1500	138.07 ± 0.11	61.78 ± 0.09
Ascorbic Acid	500	60.56 ± 0.05	27.10 ± 0.04
	1000	112.03 ± 0.09	50.13 ± 0.08
	1500	172.58 ± 0.14	77.23 ± 0.12

Values are given as mean ± standard error (three replicates).

Additionally, the TAC and IRP of free ZnO-NPs and samples doped with varying concentrations of Cu (3, 5, and 7%) increased in a dose-dependent manner. However, the antioxidant activity of the samples doped with Cu was superior to that of free ZnO-NPs at all concentrations used. The 7Cu ZnO at a concentration of 1.5 mg/mL demonstrated the highest TAC (138.07 ± 0.11 mg gallic acid/g) and IRP (61.78 ± 0.09 µg/mL). This finding is consistent with Rishikesan and Basha^53^, who demonstrated that the biological efficacy of ZnO and Cu-doped ZnO-NPs showed improved activity due to the modification of nanoparticle surfaces as a result of doping with transition metals, providing good biocompatibility and modifying chemical and physical characteristics. In comparison, at the same concentration of the standard ascorbic acid (1.5 mg/mL), the TAC and IRP were 172.58 ± 0.14 mg gallic acid/g and 61.78 ± 0.09 µg/mL, respectively.

#### Scavenging activity

**Figure 5:**
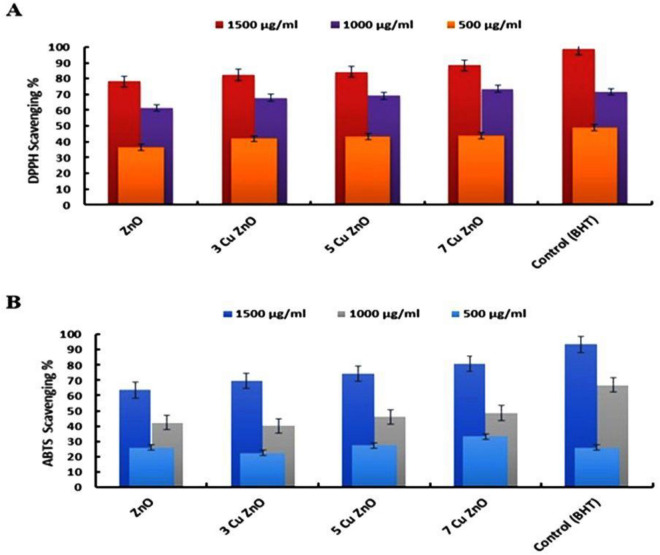
Antioxidant activity of samples using distinct methods: (**A)** DPPH scavenging activity percentage, and (**B**) ABTS% antioxidant activity of samples.

The DPPH approach is a popular method for determining antioxidant activity. DPPH molecules possess a π system, and the DPPH reagent staining method was used to measure the percentage of free radical scavenging activity of Zn and Cu^54^. The radical scavenging activity of monometallic Zn and bimetallic Cu/Zn DPPH was investigated at different dosages (500, 1000, and 1500 g/mL), with BHT serving as a control. The absorbance was restrained at 520 nm. A UV–Visible spectrophotometer was used to assess the DPPH radical scavenging potential^55^. It was observed from the results shown in Fig. 5A that the percentage inhibition of DPPH radical by bimetallic Cu/ZnO-NPs at different concentration (1500, 1000, and 500 μg/mL) was significantly higher than for monometallic ZnO-NPs and increased with increasing NPs concentration due to the synergistic effects of both metallic ingredients i.e., (82.33 ± 0.90, 67.82 ± 1.01, and 41.87 ± 0.56), (84.17 ± 0.87, 69.10 ± 0.54, and 43.12 ± 0.13) and (88.32 ± 0.33, 73.55 ± 0.76, and 44.01 ± 0.27) at (3Cu ZnO, 5Cu ZnO and 7Cu ZnO), respectively.

Also, the antioxidant capacity of 3Cu ZnO, 5Cu ZnO and 7Cu ZnO using ABTS was evaluated. Figure 5B expose that all studied assays were similarly dose-dependent, increasing progressively with increasing concentrations of bimetallic Cu/Zn. ABTS activity (%) varied from 69.54 1.02 to 80.64 ± 1.18% at 3Cu ZnO and 7Cu ZnO concentrations, respectively. Furthermore, the radical scavenging activity of ABTS increased steadily from 33.190.22 to 80.64 ± 1.18% when concentrations increased from 500 to 1500 μg/mL.

#### Anti-diabetic activity

The in-vitro biological activities of ZnO-NPs and samples doped with different concentrations of Cu (3, 5, and 7%) were tested for their anti-diabetic, anti-Alzheimer, and anti-inflammatory activities. The anti-diabetic activity was assessed by measuring the inhibitory effect against the activity of the α-amylase enzyme and comparing it to the efficiency of acarbose, a standard drug^56^. This enzyme is essential for digesting carbohydrates, and inhibiting it is a suitable way to lower postprandial hyperglycemia^57^. Data in Table 4 showed that the inhibitory effect of monometallic ZnO on the α-amylase enzyme increased with higher concentrations. The ZnO at a concentration of 1.5 mg/mL exhibited the highest inhibition percentage (31.86 ± 0.05%) compared to other concentrations (5 and 1 mg/mL). This finding is supported by Arvanag et al.^58^ and Jan et al.^59^, who reported that ZnO-NPs have a high ability to suppress α-amylase activity and exhibit strong anti-diabetic properties. The inhibitory effect of ZnO and Cu-doped ZnO-NPs on the activity of the α-amylase enzyme also increased with higher concentrations. Along with Russo et al*.*^60^, improving anti-diabetic efficacy is significantly linked to boosting antioxidant activities. Hassan and Aboulthana^61^ added that the samples with higher antioxidant activity possess stronger anti-diabetic activity.

**Table 4:** The in vitro  biological activities of ZnO-NPs and samples doped with different concentrations of Cu (3, 5, and 7%).

Sample	Concentration (µg/mL)	Anti-diabetic activity	Anti-alzheimer activity	Anti-inflammatory activity
		α-amylase	AChE	Proteinase
		Inhibition (%)		
ZnO	500	11.18 ± 0.02	12.25 ± 0.03	10.76 ± 0.02
	1000	20.68 ± 0.03	12.43 ± 0.04	10.92 ± 0.02
	1500	31.86 ± 0.05	12.48 ± 0.04	10.96 ± 0.02
3Cu ZnO	500	13.98 ± 0.02	12.43 ± 0.04	10.92 ± 0.02
	1000	25.85 ± 0.04	12.65 ± 0.04	11.11 ± 0.02
	1500	39.83 ± 0.06	12.66 ± 0.04	11.12 ± 0.02
5Cu ZnO	500	17.19 ± 0.03	12.46 ± 0.04	10.94 ± 0.02
	1000	31.80 ± 0.05	12.49 ± 0.04	10.96 ± 0.02
	1500	48.99 ± 0.08	12.49 ± 0.04	10.97 ± 0.02
7Cu ZnO	500	21.49 ± 0.03	12.49 ± 0.04	10.97 ± 0.02
	1000	39.75 ± 0.06	12.52 ± 0.04	10.99 ± 0.02
	1500	61.24 ± 0.09	12.60 ± 0.04	11.06 ± 0.02

Sample	Concentration (µg/mL)	Anti-diabetic activity	Anti-alzheimer activity	Anti-inflammatory activity
		Acarbose	Donepezil	Diclofenac sodium
		Inhibition (%)		
STD	500	26.86 ± 0.04	28.11 ± 0.08	24.68 ± 0.05
	1000	49.69 ± 0.08	52.00 ± 0.15	45.66 ± 0.10
	1500	76.55 ± 0.12	80.11 ± 0.23	70.35 ± 0.15

Values are given as mean ± standard error (three replicates).

**Figure 6:**
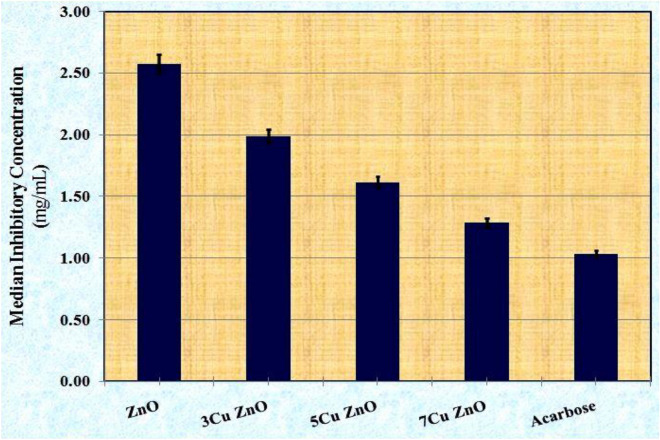
The median inhibitory concentrations (IC_50_) of ZnO-NPs and samples doped with different concentrations of Cu (3, 5 and 7%) against activity of α-amylase enzyme compared to Acarbose as a standard.

**Figure 7:**
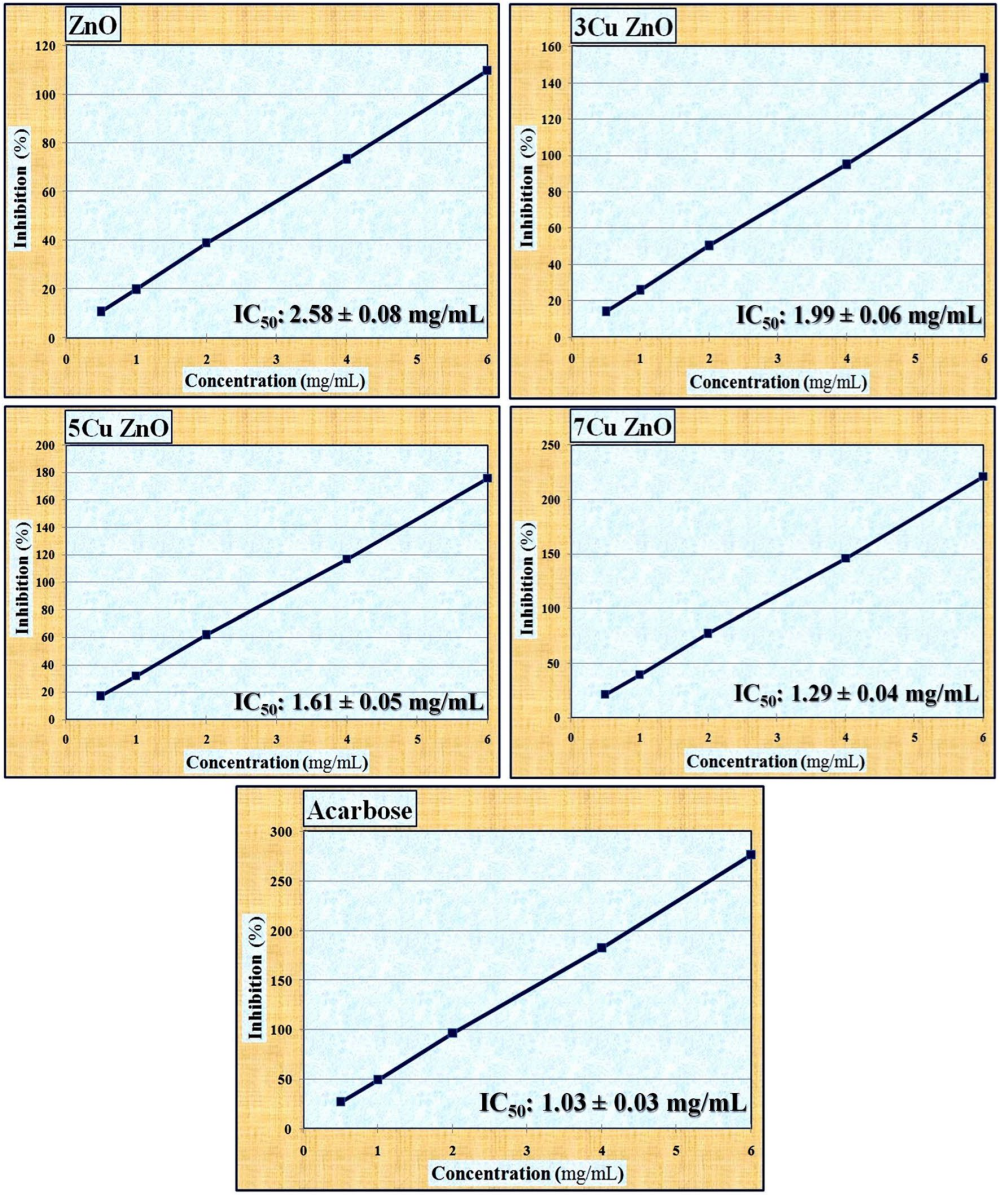
The curves used for calculating the median inhibitory concentrations (IC_50_) of ZnO-NPs and samples doped with different concentrations of Cu (3, 5, and 7%) against  the activity of the α-amylase enzyme compared to Acarbose as a standard.

The data presented in Fig. 6 illustrates the IC_50_ values of free ZnO-NPs and samples doped with varying concentrations of Cu (3, 5, and 7%) in comparison to the acarbose standard. The curves used for determining the IC_50_ values of all the studied samples are shown in Fig. 7. The concentration of the ZnO sample needed to impede 50% of the α-amylase activity was approximately 2.58 ± 0.08 mg/mL. This finding is consistent with Abbasi et al.^62^ and further supported by Aboulthana et al.^63^, who emphasized that ZnO-NPs have the ability to inhibit α-amylase activity, with a low IC_50_ indicating higher anti-diabetic activity. The IC_50_ values of free ZnO-NPs and samples doped with different concentrations of Cu (3, 5, and 7%) decreased in a dose-dependent manner, indicating that the anti-diabetic activity of the samples doped with Cu was superior to the antioxidant activity of free ZnO-NPs at all concentrations used. The 7Cu ZnO at a concentration of 1.5 mg/mL exhibited the highest inhibitory effect on α-amylase activity (61.24 ± 0.09%) and consequently the lowest IC_50_ value (1.29 ± 0.04 mg/mL). In comparison, at the same concentration of the standard acarbose (1.5 mg/mL), the inhibition percentage on α-amylase enzyme and its IC_50_ value were 76.55 ± 0.12% and 1.03 ± 0.03 mg/mL, respectively. Acarbose standard is a commercially used enzyme inhibitor that demonstrates significantly high inhibition values at the highest concentrations. However, it is expensive and commonly associated with side effects in its applications^64^.

**Figure 8:**
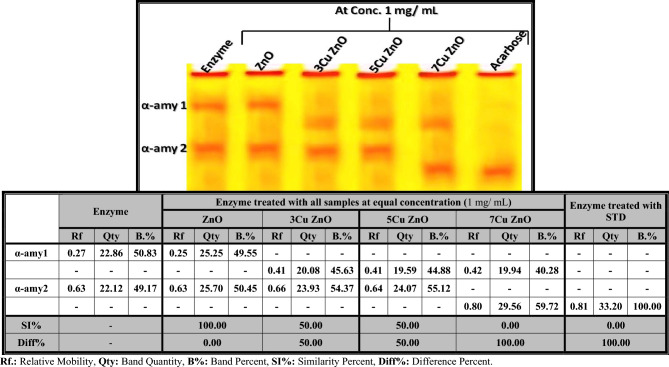
Native electrophoretic α-amylase isoenzyme pattern showing the effect of zinc oxide nanoparticles (ZnO-NPs) and samples doped with different concentrations of Cu (3, 5, and 7%) on the physiological state of α-amylase enzyme compared to Acarbose as a standard.

As shown in Fig. 8, it was observed that the standard α-amylase enzyme was electrophoretically represented by 2 types identified at Rfs 0.27 and 0.63 (Qty 22.86 and 22.12; B% 50.83 and 49.17, respectively). The standard enzyme was treated with an equal concentration (1 mg/mL) of ZnO-NPs and samples doped with different concentrations of Cu (3, 5, and 7%). The crude ZnO caused no electrophoretic variation in the α-amylase pattern. Therefore, the electrophoretic α-amylase pattern is completely similar to the standard enzyme (SI = 100.00%; Diff. = 0.00%). Treatment of the standard enzyme with the samples doped with the concentrations 3 and 5% of Cu caused the same adverse effect on the electrophoretic isoenzyme pattern, signified by hiding the normal α-amy 1 with the presence of one abnormal band identified at Rf 0.41 with Qty 20.08 and B.% 45.63 with 3Cu ZnO and first (3, 5, and 7%) with Qty 19.59 and B.% 44.88 with 5Cu ZnO. Therefore, the electrophoretic α-amylase pattern in both 3Cu ZnO and 5Cu ZnO is identical to the standard enzyme by 50.00% (SI = 50.00%; Diff. = 50.00%). Regarding 7Cu ZnO, it was found that it caused severe deleterious effect on the standard enzyme represented by hiding all normal α-amylase types with the existence of 2 abnormal ones identified at Rfs 0.42 and 0.80 (Qty 19.94 and 29.56; B.% 40.28 and 59.72, respectively). This is in agreement with Agarwal and Henkin^65^ and supported by Hong et al*.*^66^, who postulated that α-amylase has metal-binding capacities like Cu^2+^ and Zn^2+^. Martins et al*.*^67^ discovered that α-amylase contains two metal-binding sites, one for Ca^2+^ only and the other for Cu^2+^ plus Fe^3+^. These studies confirm our findings, indicating that Cu^2+^ has a strong interaction with this enzyme.

The Cu^2+^ ions were found to be present both on the surface and inside the Cu-doped ZnO nanocrystals. The ions situated on the surface of the nanocrystals facilitated interaction with specific biomolecules, promoted mass transfer and electron transfer, and helped to prevent photo-corrosion of the nanocomposites, ultimately enhancing their efficiency^68^. The most severe effect observed in the electrophoretic α-amylase pattern was caused by the standard (acarbose), which resulted in abnormalities characterized by the absence of normal α-amylase types and the presence of only one abnormal band identified at Rf 0.81 (Qty 33.20; B.% 100.00). As a result, the electrophoretic α-amylase pattern in both 7Cu ZnO and acarbose standard is completely different from the standard enzyme (SI = 0.00%; Diff. = 100.00%).

#### Anti-Alzheimer's activity

Inhibition of the AChE enzyme is an effective strategy for treating Alzheimer’s disease because the activation of this enzyme is a leading cause of the disease^69^. In the current experiment, it was observed that both free ZnO-NPs and samples doped with different concentrations of Cu (3%, 5%, and 7%) did not have a significant inhibitory effect and did not cause any variation in AChE activity at all studied concentrations (5 mg/mL, 1 mg/mL, and 1.5 mg/mL). In contrast, at the same concentrations, the standard donepezil showed inhibitory effects of 28.11 ± 0.08, 52.00 ± 0.15, and 80.11 ± 0.23, respectively (Table 4).

#### Anti-inflammatory activity

Inflammation is considered the most common phenomenon induced by protein denaturation^70^. The anti-inflammatory activity was assessed by measuring the efficiency of the tested substance in inhibiting the activity of the proteinase enzyme^71^. Recently, Ayman et al.^72^ demonstrated that inhibition of this enzyme indicates potential for anti-inflammatory activity. Regarding the data on anti-inflammatory activity obtained during the current study (Table 4), it was observed that free ZnO-NPs and samples doped with different concentrations of Cu (3, 5, and 7%) did not cause significant differences in the activity of the proteinase enzyme among all studied concentrations (5, 1, and 1.5 mg/mL). In contrast, at the same concentrations, the standard Diclofenac Sodium showed values of 24.68 ± 0.05, 45.66 ± 0.10, and 70.35 ± 0.15, respectively. Therefore, the current experiment revealed that free ZnO-NPs and samples doped with different concentrations of Cu (3, 5, and 7%) showed no anti-Alzheimer and anti-inflammatory activities. Further studies are recommended to be carried out in the future to evaluate the efficiency of free ZnO-NPs and samples doped with Cu in streptozotocin-induced diabetes in rats and compare them to commercially available anti-diabetic standard drugs.

Table 5 shows that the TAC, IRP, and inhibition % of α-amylase are positively correlated with each other at equal concentrations (1 mg/mL) of the studied samples, with a significance level of P ≤ 0.05. It was observed that as antioxidant activities increase, so does the anti-diabetic activity. This supports the findings that the inhibitory effect against α-amylase enzyme is strongly associated with increasing antioxidant efficiency. However, the inhibitory effect against AChE and proteinase enzymes did not show significant correlation with other in vitro biological activities.

**Table 5:** The statistical correlations among the different in-vitro biological activities of ZnO-NPs and samples doped with different concentrations of Cu (3, 5, and 7%).

	TAC	IRP	Inhibition (%)		
			α-Amylase	AChE	Proteinase
TAC	–	0.00**	0.00**	0.85	0.84
IRP	0.00**	–	0.00**	0.84	0.84
Inhib. AChE					
α-Amylase	0.00**	0.00**	–	0.84	0.84
AChE	0.85	0.84	0.84	–	0.07
Proteinase	0.84	0.84	0.84	0.07	–

**The correlation is significant at *P* ≤ 0.05.

#### Antimicrobial activity

**Figure 9:**
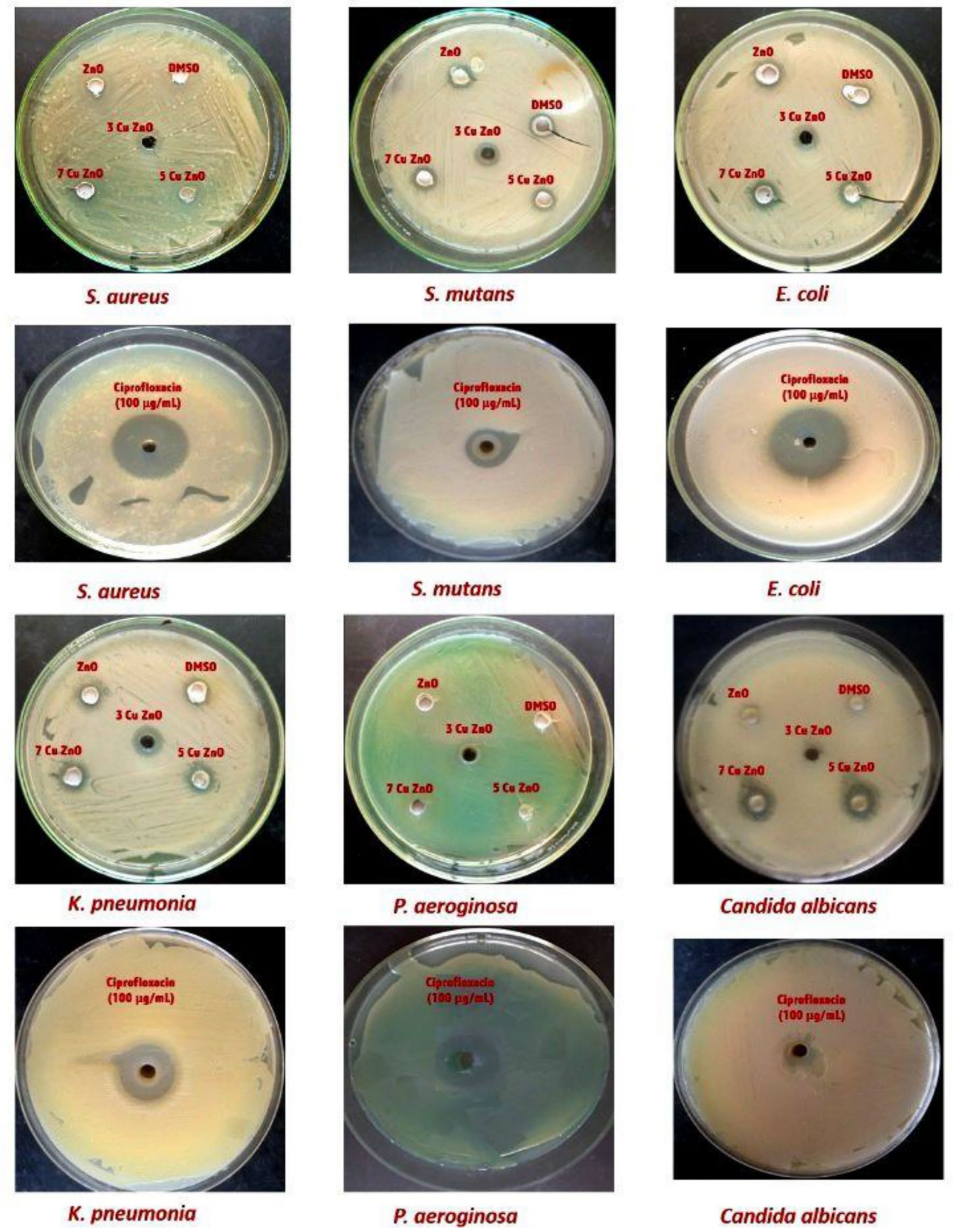
Antimicrobial activity of samples against *E. coli, Staphylococcus aureus, P. aeroginosa, Streptococcus mutants*, *K. pneumonia,* and *Candida albicans*.

Alternative drug development is required to combat antibiotic-resistant microorganisms. Metal oxide nanocomposites could be one of the options for fighting numerous microbial infections^73^. As shown in Fig. 9 and Table 6, copper-doped zinc oxide NPs have the highest antibacterial activity while ZnO-NPs have the lowest against diverse bacteria. The cupper-doped ZnO-NPs were tested at different concentrations of 3%, 5%, and 7% for their inhibitory effects against specific bacteria. The results showed that the NPs exhibited a moderate level of inhibition with varying zone sizes in the respective wells. For *E. coli*, the zone of inhibition observed was 4.5 ± 0.1, 5.0 ± 0.1, and 5.5 ± 0.1 at concentrations of 3, 5, and 7%, respectively compared with Ciprofloxacin 7.50 ± 0.2. Similarly, for *K. pneumonia*, the zones of inhibition were measured as 7.3 ± 0.3, 7.5 ± 0.3, and 7.5 ± 0.3 mm at the same concentrations compared with Ciprofloxacin 6.50 ± 0.0 mm.

**Table 6:** Antimicrobial activity of samples evaluated by well diffusion method.

Microorganisms	Type	Antimicrobial activity (mm)					
		DMSO	1000 μg/mL				Ciprofloxacin (100 μg/mL)
			ZnO	3Cu ZnO	5Cu ZnO	7Cu ZnO	
*S. aureus*	G + ve	Nil	2.0 ± 0.1	2.5 ± 0.1	2.5 ± 0.1	2.5 ± 0.1	8.30 ± 0.2
*S. mutans*	G + ve	Nil	3.5 ± 0.2	4.0 ± 0.2	4.5 ± 0.2	4.5 ± 0.2	5.50 ± 0.1
*E. coli*	G − ve	Nil	2.8 ± 0.1	4.5 ± 0.1	5.0 ± 0.1	5.5 ± 0.1	7.50 ± 0.2
*K. pneumonia*	G – ve	Nil	4.3 ± 0.3	7.3 ± 0.3	7.5 ± 0.3	7.5 ± 0.3	6.50 ± 0.0
*P. aeruginosa*	G – ve	Nil	0.0 ± 0.0	0.0 ± 0.0	0.0 ± 0.0	0.0 ± 0.0	11.20 ± 0.0
*Candida albicans*	–	Nil	0.0 ± 0.0	2.5 ± 0.1	7.4 ± 0.3	8.0 ± 0.2	Nil

The experiment was repeated three times. The values are reported as mean standard deviation.

In contrast, the zone of inhibition for *S. aureus* was measured as 2.5 ± 0.1, 2.5 ± 0.1, and 2.5 ± 0.1 mm at concentrations of 3, 5, and 7%, respectively compared with Ciprofloxacin 8.30 ± 0.2 mm. Similarly, for *S. mutans*, the zones of inhibition were observed as 4.0 ± 0.2, 4.5 ± 0.2, and 4.5 ± 0.2 mm at concentrations of 3, 5, and 7%, respectively compared with Ciprofloxacin 5.50 ± 0.1 mm. Also, it produced the highest zone of inhibition with 7.4 ± 0.3, and 8.0 ± 0.2 mm for *C. albicans* at 5, and 7% of copper-doped ZnO-NPs, respectively. Also, no inhibition was observed for *P. aeruginosa* while (Ciprofloxacin 11.20 ± 0.0 mm). In addition, the inhibitions of only ZnO-NPs without cupper were 2.0 ± 0.1, 3.5 ± 0.2, 2.8 ± 0.1, and 4.3 ± 0.3 mm for *S. aureus*, *S. mutants, E. coli*, and *K. pneumonia*, respectively. Hence, based on our results the copper-doped ZnO-NPs possess promising antimicrobial activities against the studied pathogens. The results of this study are consistent with previous research conducted by El-Borady and El-Sayed^74^ who reported the conjugations of ZnO-NPs with folic acid (FA), may achieve more antimicrobial activity. In addition, El-Borady et al.^75^ investigated the antibacterial properties of three types of ZnO-NPs against four pathogenic bacterial strains. Metal oxide nanoparticles can overcome microbial drug resistance and operate as effective antibacterial agents by disrupting bacterial cell membranes^76^. Furthermore, the inhibitory effect of ZnO, Ag/ZnO, CuO, and Ag/CuO nanocomposites on bacterial growth is thought to result from the formation of reactive oxygen species (ROS), for example superoxide anion (O^2−^), hydrogen peroxide (H_2_O_2_), hydroxyl radicals (HO%) and organic hydroperoxides (OHP)^77,78^. These ROS can physically disrupt bacterial cell membranes, causing proteins and lipids to collapse. Copper ions may adhere to and shatter the negatively charged bacterial cell wall in the case of CuO and Ag/CuO-NPs, resulting in protein denaturation and cell death^79^.

### Molecular docking simulation

**Figure 10:**
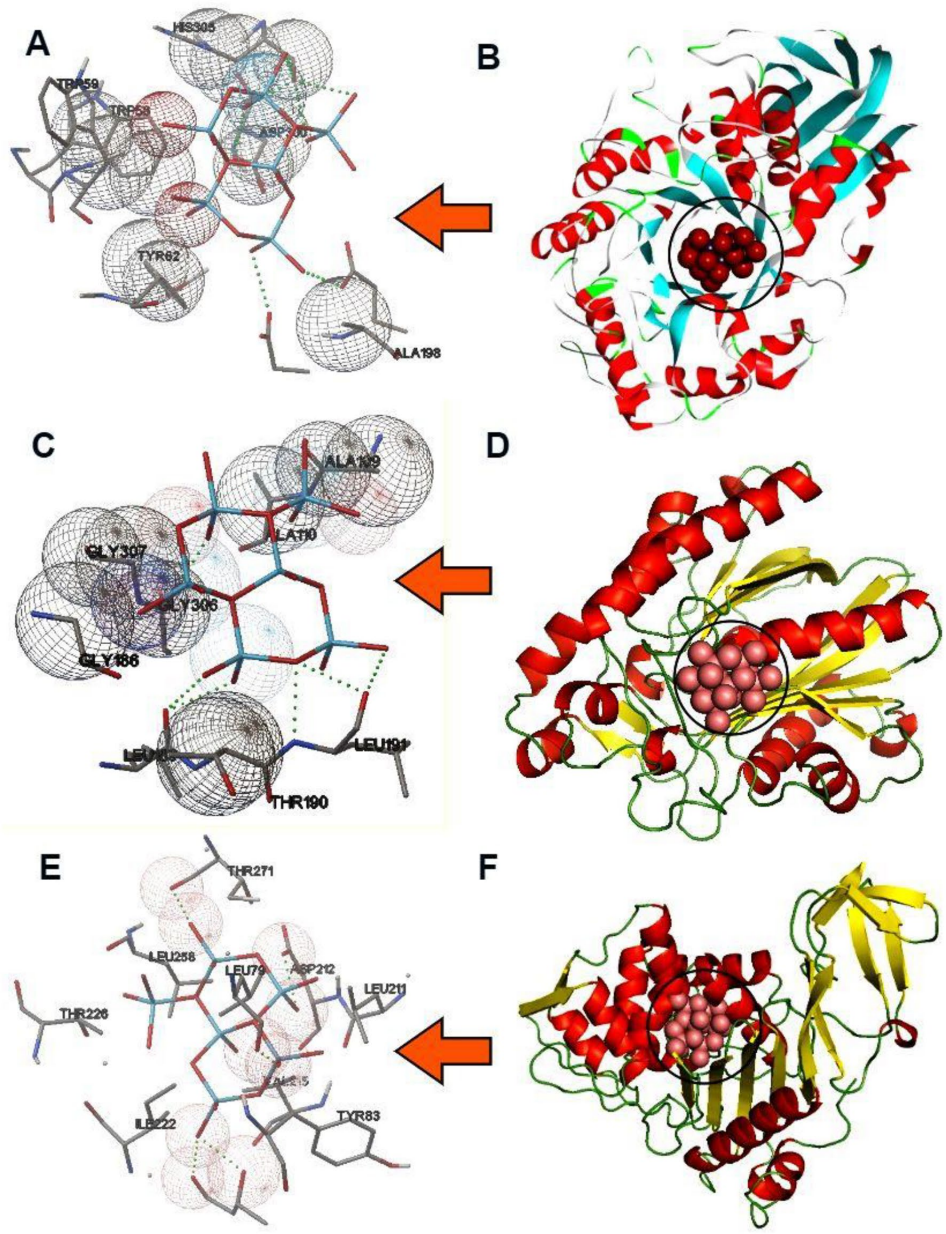
3D representations of Cu-doped ZnO conformations and interaction between common residues at the binding pocket: (A and B) α-amylase (PDB: 2QV4), (C and D) FabH of *E. coli* (PDB: 5BNM), and (E and F) Penicillin binding protein of *S. aureus* (PDB: 3HUM).

We performed molecular docking, which allowed us to investigate the binding interactions between Cu-doped ZnO and protein targets involved in anti-diabetic and anti-microbial activities, supporting their efficacy. The docking results between Cu-doped ZnO and the anti-diabetic enzyme α-amylase indicate a strong affinity of − 8.45 kcal/mol (Table 7). The interaction is facilitated by the formation of eight hydrogen bonds with Ala198, His305, Trp59, Trp58, Asp300, Tyr62, Asp197, and Glu233. Additionally, hydrophobic contacts were formed, including three carbon-hydrogen bonds with His305 and Gly306, and three attractive bonds with Asp300, Asp197, and Glu233. The common residues His305, Ala198, and Glu233 in the catalytic site contribute to enhancing the binding affinity. Figure 10A and B displays the docked poses of Cu-doped ZnO and α-amylase, providing a visual representation of their binding interaction. According to our docking, the key residues involved in the interaction between Cu-doped ZnO and α-amylase are Ala198, His305, Trp59, Trp58, Asp300, Tyr62, Asp197, and Glu233. These residues likely play a crucial role in stabilizing the Cu-doped ZnO-α-amylase complex and mediating specific interactions between Cu-doped ZnO and α-amylase. This agrees with Melk et al.^80^, who used the molecular docking method to evaluate the inhibitory interaction between a small molecule and α-amylase. Additionally, the anti-microbial target FabH is an enzyme involved in the fatty acid synthesis pathway in *E. coli*. According to the docking results, Cu-doped ZnO has an affinity of -7.89 kcal/mol, compared to Ciprofloxacin (− 7.80 kcal/mol). Cu-doped ZnO formed nine hydrogen bonds with Ala111, Leu191, Gly306, Leu189, Gly186, Gly183, Gly307, and Thr190 (Fig. 10C and D). Also, hydrophobic contacts included one Metal-Acceptor bond with Leu191. The frequent residues Arg90, Arg80, Trp30, and Val77 at the catalytic site are found to improve binding affinity. Our docking analysis revealed that the essential residues involved in the interaction between Cu-doped ZnO and FabH are Ala111, Leu191, Gly306, Leu189, Gly186, Gly183, Gly307, and Thr190. These residues in the catalytic region were found to increase the binding affinity of Cu-doped ZnO with the FabH enzyme. Our in-silico findings indicate that Cu-doped ZnO exhibits a stronger binding affinity for the FabH enzyme^81^. These results are consistent with Jawhari et al.^82^, who identified and synthesized compounds with potential as FabH inhibitors. Furthermore, Penicillin-binding proteins (PBPs) are a group of enzymes found in *S. aureus* that play a crucial role in cell wall synthesis. According to the docking findings, Cu-doped ZnO exhibits binding energies of − 7.90 kcal/mol for Penicillin-binding protein, compared to Ciprofloxacin (− 7.10 kcal/mol). Additionally, seven hydrogen bonds were formed with Thr219, Lys259, Thr271, Leu211, Asp212, Tyr83, and Tyr208. Two carbon-hydrogen bonds with Leu79 and Gly257 were also formed (Fig. 10 (E and F)). Moreover, it can be perceived that the key residues Lys259, Leu211, and Asp212 in the catalytic site improve the binding affinity. Our findings are consistent with Khidre et al.^83^, who also employed docking to evaluate the inhibitory interaction between a molecule and Penicillin-binding protein. Additionally, Rashid et al.^84^ used molecular docking to highlight the binding interactions of Cu-doped ZnO with β-lactamase and FabH enzyme targets.

**Table 7:** Molecular interactions of Cu-doped ZnO with amino acids of anti-diabetic and anti-microbial target proteins

	Ligands	Proteins	3D structure	Hydrophilic interactions		Hydrophobic contacts		No. of H-bonds	No. of Total bonds	affinity kcal mol-1
				Residue (H- Bond)	Length	Residue (bond type)	Length			
1	Cu-doped ZnO nanocrystals	alpha-amylase		Ala198 (H- Bond) His305 (H- Bond) Trp59 (H- Bond) Trp58 (H- Bond) Asp300(H- Bond) Tyr62(H- Bond) Asp197(H- Bond) Glu233(H- Bond)	3.45 3.18 2.33 2.65 3.55 2.44 3.55 2.92 3.14	His305, (Carbon-H-Bond) Gly306, (Carbon -H-Bond) Gly306, (Carbon -H-Bond) Asp300, (Attractive Charge) Asp197, (Attractive Charge) Glu233, (Attractive Charge)	2.82 3.22 2.92 4.57 3.40 5.44	**8**	**14**	− **8.45**
2		FabH of *E. coli*		Ala111 (H- Bond) Leu191 (H- Bond) Gly306 (H- Bond) Leu191(H- Bond) Leu189(H- Bond) Gly186(H- Bond) Gly183(H- Bond) Gly307(H- Bond) Thr190(H- Bond)	3.19 2.64 2.70 2.49 2.55 3.29 3.08 2.93 3.12	Leu191, (Metal-Acceptor)	3.26	**9**	**10**	− ** 7.89**
3		Penicillin binding protein of *S. aureus*		Thr219 (H- Bond) Lys259 (H- Bond) Thr271 (H- Bond) Leu211(H- Bond) Asp212(H- Bond) Tyr83(H- Bond) Tyr208(H- Bond)	2.53 2.25 2.99 2.92 2.57 4.14 3.80	Asp212, (Attractive Charge) Asp212, (Attractive Charge) Leu79 (Carbon -H-Bond) Gly257 (Carbon -H-Bond)	3.86 4.88 3.73 2.35	**7**	**11**	− **8.55**

Ala, Alanine; Asn, Asparagine; Asp, Aspartic acid; Glu, Glutamic acid; Gln, Glutamine; Gly, Glycine; His, Histidine; Leu, Leucine; Lys, Lysine; Thr, Threonine; Trp, Tryptophan; Tyr, Tyrosine.

## Conclusion

This study presents an investigation of the microstructural, antioxidant, and antimicrobial properties of Cu-doped ZnO [ZnO_1−x_ Cu_x_O: _x_ = 0.0, 0.03, 0.05, and 0.07] nanoparticles. The standard chemical co-precipitation procedure was used. P-XRD confirmed the generation of pristine hexagonal wurtzite crystallographic phase for all samples. Because Cu^2+^ ions and Zn^2+^ ions have similar ionic radii of 7.3 and 7.4 nm, respectively, the XRD study revealed that the doping of Cu^2+^ ions into the ZnO crystal had minor effects on the lattice characteristics of ZnO (a and c). HR-TEM micrographs of ZnO and Cu_2_O show a definite boundary in the images, indicating a strong link between them. The study demonstrated that Cu-doped ZnO possess significant scavenging activity against DPPH and ABTS radicals, indicating its ability to neutralize free radicals and protect against oxidative stress. Moreover, Cu-doped ZnO exhibited superior antimicrobial efficacy against pathogenic microorganisms compared to pure ZnO, likely due to the presence of copper ions, which enhanced its antimicrobial properties. These combined results suggest the potential utility of Cu-doped ZnO in various biomedical applications. Additionally, molecular docking simulations highlighted that Cu-doped ZnO displayed lower binding energy at the active sites of α-amylase, and antimicrobial receptors indicating its potential inhibitory effect on these enzymes. Therefore, Cu-doped ZnO nanoparticles show promise for combating diabetes and microbial infections.

## Data Availability

All data generated or analyzed during this study are included in this published article.
